# A Multi-Disciplinary Approach to Intimate Partner Violence: A Qualitative Study of the Perpetrators’ Experiences

**DOI:** 10.1177/08862605251355622

**Published:** 2025-07-15

**Authors:** Camilla Buch Gudde, Tom Palmstierna, Richard Whittington, Merete Berg Nesset

**Affiliations:** 1 St. Olav’s Hospital, Trondheim University Hospital, Norway; 2 Norwegian University of Science and Technology (NTNU), Faculty of Medicine and Health Sciences, Trondheim, Norway; 3 Centre for Psychiatric Research, Karolinska Institutet, Stockholm, Sweden

**Keywords:** intimate partner violence, perpetrator, early intervention, multi-disciplinary, qualitative research

## Abstract

High-risk perpetrators of intimate partner violence (IPV) have high drop-out rates from treatment and frequently recidivate into violence towards the partner, despite efforts by services to help the person to change their behavior. The current body of scientific evidence is still too small to conclude about the effectiveness of perpetrator interventions in ending violence. There is a growing interest among researchers and agencies in exploring multi-disciplinary approaches to IPV. In accordance with this, it is essential to ask high-risk perpetrators of IPV about their needs in order to stop acting violently. Hence, the aim of this study was to explore the perpetrators’ perspectives on the violence risk-reducing interventions implemented by the police and the health- and social services. We conducted 13 semi-structured, individual interviews with high-risk perpetrators of IPV (11 male) who were referred to a multidisciplinary treatment team. The interviews were analyzed according to the Systematic Text Condensation method. Most of the participants experienced a critical period after being reported to the police, with feelings of being left alone in a state of practical and emotional distress without sufficient support. Further, they elaborated on their experiences of receiving help and support from the early onset multidisciplinary team. They emphasized the experience of being met with a holistic approach and regarded as a human being with anger problems rather than just a perpetrator. Finally, they identified their treatment needs in order to desist violence, and highlighted the importance of early and easily accessible support after the actual offence. The findings highlight the necessity of providing hope and easy access to immediate and multi-disciplinary interventions in order to prevent recurrent IPV. The police as well as the health- and social services have a unique opportunity to act in a manner that may reduce the risk of repeated IPV.

## Background

Intimate partner violence (IPV) is a global issue seriously affecting the mental health, wellbeing, and physical health of both victims and perpetrators throughout the life course (Oram et al., 2022; WHO, 2021). In Norway, the socio-economic costs of violence in close relationships were estimated to be 92.7 billion NOK in 2021 (Pedersen et al., 2023). Violence between parents has been linked to violence against children and youth (Syed et al., 2023). A Norwegian cross sectional study of youth aged 12 to 16 years reported that 18% had witnessed violence against their mother and 14% against their father (Hafstad & Augusti, 2019).

The likelihood of perpetrating IPV is linked to adverse life circumstances (Breet et al., 2019). On the individual perpetrator level, being exposed to adverse experiences in early childhood, such as witnessing or being subjected to domestic violence, will negatively affect the person’s mental health and may impair their ability to cope with stressful life circumstances in adulthood (Petruccelli et al., 2019; Syed et al., 2023). Furthermore, cognitive distortions, problems with emotion regulation, and low self-esteem have been found to negatively affect the capacity for change processes among perpetrators undergoing treatment (McGinn et al., 2020). A population-based longitudinal study reported a clear association between a number of mental health disorders and an increased risk for perpetrating IPV (Yu et al., 2019). These findings are supported by a number of studies which have identified a relationship between post-traumatic stress disorder, depression, and anxiety with perpetration of IPV by both males and females (Breet et al., 2019; Capaldi et al., 2012; Oram et al., 2014; Tutty et al., 2020). Interventions intended to reduce violent behavior have yielded mixed effects and to date there is a question of what works for whom under which circumstances (Karakurt et al., 2019; Oğuztüzün et al., 2023). Moreover, attrition is a major issue among intimate partner violent perpetrators undergoing treatment, especially among those with more generally violent propensities (Lila et al., 2019; Petersson & Strand, 2017). A meta-analysis examining drop-out among individuals undergoing perpetrator intervention programs found drop-out rates between 9% and 67% (Cunha et al., 2024). Problems with substance use and mental health have been shown to be associated with drop-out from treatment (Lila et al., 2019), and research on treatment drop-out and violence recidivism has suggested that those who would potentially benefit the most from treatment are the very ones who are least likely to complete it (Cunha et al., 2024; Olver et al., 2011).

In order to tailor interventions it is meaningful for the police and health- and social services to first adequately assess an individual’s risk in relation to specific violence characteristics, the types and severity of violence, the target, and the circumstances (Oğuztüzün et al., 2023; Travers et al., 2021). The assessment of risk for IPV has been described as the process of gathering information about people to make decisions on their risk of perpetrating IPV (Kropp & Hart, 2015). It is pivotal for the police and health- and social services to investigate and intervene early when identified IPV risk factors are present as these may, in a worst-case scenario, end in intimate partner murder (Garcia-Vergara et al., 2022; Juodis et al., 2014; C. M. Spencer & Stith, 2020; C. M. Spencer et al., 2022). However, police practices when responding to the perpetrators’ risks and needs after being reported will depend on the police officers’ understanding of the complex dynamics of IPV (Gill et al., 2021). Moreover, the police officers’ definition of what constitutes violence (i.e., physical attacks) will influence their assessment of the risks for further violence and the needs of both the perpetrator and the victim (Nesset et al., 2020; Serrano-Montilla et al., 2023). Previous studies have suggested that some perpetrators are more generally violent (i.e., they are violent to other people as well as their intimate partner) and such perpetrators are more prone to recidivate in their IPV, despite services’ efforts to prevent the violence from occurring (Cunha et al., 2024; Lila et al., 2019; Petersson & Strand, 2017).

The complexity that characterizes violent intimate partnerships makes it especially challenging for the health- and social services and the police to intervene effectively (Oram et al., 2022), and researchers have highlighted that multidisciplinary partnership working with representatives from the police and the health- and social services altogether is a novel and potentially more effective way to address the global epidemic of violence in close relationships (Gani & Chandan, 2023; Heise, 1998; Juodis et al., 2014; NICE, 2014; WHO, 2020). Accordingly, there is an urgent need to rethink existing approaches to domestic violence, and focus on multi-agency interventions for the victims and perpetrators simultaneously (Expósito-Álvarez et al., 2023; Gani & Chandan, 2023).

### The High-Risk Team

To be able to work effectively with perpetrators at high risk of recurrent and severe IPV, professionals from the police, community services, and specialist health services in Trondheim, Norway, created an entity known as the High-Risk Team. The three agencies signed a mutual agreement, committing them to collaborate in high-risk cases of IPV. The goal of this multidisciplinary team was to intervene according to the victim and perpetrator’s individual needs and to offer services to the whole family conjointly.

In this team, specialist police officers make an initial risk assessment of all IPV incidents using the Brief Spousal Assault Form for the Evaluation of Risk (B-SAFER; Kropp et al., 2005). If the case is assessed as presenting a high risk of severe violence, the couple are offered help from the High-Risk Team. The perpetrators and the victims in each event provide written consent that allows the team to share relevant information about dynamic risk and the couple’s health- and social needs between team members from different agencies. When a new case is referred to the team, a joint decision on the risk level and appropriate measures towards the victims (adults and children) and the perpetrator is made. Allocated team members offer help and support to both the victims and the perpetrator respectively. Professionals from a victim support center provides information about the legal process, as well as psychosocial support to the adult victim until a verdict is reached. Furthermore, they provide guidance and information about how to report a crime and help the victim to apply for criminal injury compensation. The Children’s House focuses on the children in the family. Their goal is to help the children when the parents are suspected of domestic violence, and to provide psychological first aid to the child. Therapists from the specialist health services and the community assess the perpetrator’s dynamic violence risk, suicidality and needs, as well as offering emotion regulation. Finally, they refer the perpetrator to other adequate health- and social services for more long-term follow-up.

The time in contact depends on the level of risk in the actual case at hand. Regular contact between the key workers (by phone and physical meetings) makes it possible to monitor dynamic changes in violence risk in each case.

Given the novelty of this approach it is important to explore the views and needs of key stakeholders, including perpetrators, but there is sparse research using a qualitative design to do this (McGinn et al., 2020). Such studies could potentially inform the planning and design of risk reducing interventions. In light of the aforementioned challenges, it is essential to ask high-risk perpetrators of IPV about their needs, which, if addressed, might stop them acting violently. Hence, the aim of this study was to explore the perpetrators’ perspectives on current violence risk-reducing interventions implemented by the police and the health- and social services, and to identify scope for improving the provision of such interventions.

## Method

Given the limited amount of research in this field, we chose a qualitative method in order to obtain rich descriptions of the IPV perpetrators’ experiences and opinions without any pre-existing agenda or assumptions. A phenomenological approach was therefore adopted, a methodology that focuses on first-hand experiences as the source of knowledge, as they were lived by the participants (Smith, 2024). We collected data for this purpose through semi-structured, individual interviews. The research team consisted of researchers with experience from the field of intimate partner violence and qualitative research. There was no relationship between the interviewer and the participants prior to the study commitment.

### Participants and Recruitment

The participants for the study were recruited from referrals to the High-Risk Team. Psychiatric nurses and social workers performed an initial clinical assessment of eligibility to participate in the research study. Reasons for not offering perpetrators the opportunity to participate in the study were typically ongoing drug misuse, mental instability or if they had moved away, that is, to another part of the country or abroad.

A brief preliminary information about the study was provided by the therapists in the High-Risk Team, who also obtained verbal permission to forward contact information to the researcher. Of the 31 eligible perpetrators contact information for 17 individuals was provided to the researchers. One of the researchers (C.B.G.) contacted the participants, gave further information about the study, and asked for written consent to participate. Out of the 17 individuals, 4 did not answer or declined the invitation. The final sample constituted of 13 individuals and represented diversity regarding sex, age, and mother tongue, as well as socio-economic characteristics like education and work status (see Table 1).

**Table 1.** Characteristics of Participants (*n* = 13 Perpetrators).

Characteristics	*n*
Gender	
Male	11
Female	2
Age	
20–29	4
30–39	2
40–49	5
50–59	2
Levels of education	
Primary school	1
High school	11
College/University up to 3 years	1
Relationship to partner^a^	
In a relationship	8
Terminated relationship	4
Children in the household	
No	8
Yes, live with their own/partner’s children	5
Employment^b^	
Full time or part time (50%–100%)	7
On sick leave/benefits	6
Mother tongue	
Norwegian	10
Other	3

a Missing data in one case.

b At the time of the interview.

### Data Collection

Information regarding gender, age, mother tongue, educational level, working situation, and relationships to the partner and children in the household was collected on a demographic form. To explore the participants’ experiences, we performed semi-structured individual interviews, allowing the participants to provide rich descriptions of their experiences in their own words. The procedure allowed any themes to emerge as these were not pre-determined by the researchers. The interviews were based on a semi-structured interview guide with five main topics related to the participant’s experiences of the initial meeting with the police, the general health and social services, and the multidisciplinary High-Risk Team (Additional file 1—Supplemental Material). The initial topic guide was adjusted throughout the study, and statements from initial participants were followed up with subsequent interviewees exploring if others shared the same experience.

The interviews were conducted between February 2019 and August 2021 and lasted from 23 to 102 min (mean: 61 min, median: 55 min). One of the authors (C.B.G.) performed the interviews, which might have reduced the potential for interviewer effect response bias. All interviews were conducted in Norwegian, audio-recorded and transcribed verbatim. The participants were interviewed once, and all interviews were conducted in conjunction with appointments with the High-Risk Team. The interviewer checked the audio file immediately after each interview to ensure if clarifications were needed.

### Analysis

In line with the descriptive and explorative design of the study, the data were analyzed following the Systematic Text Condensation method (STC), a thematic cross-case analysis approach inspired by Giorgi’s phenomenological analysis and modified by Malterud (Malterud, 2001, 2012). STC is a four-step analysis procedure with ambitions to describe the participants’ experiences as they express them.

The first step involved all the transcripts being read in full in order to obtain an overall impression of the material, meeting the data open minded with awareness to the participants’ voices. In the second step, the transcripts were systematically reviewed, line by line, in order to identify and code meaning units relevant for the research questions and the phenomenon being studied. The meaning units were coded and sorted underneath preliminary themes. Corresponding codes were sorted into code groups. Thereafter, the preliminary themes were reduced to three distinct themes with associated sub-themes, based on iterative discussions among the authors. The third step involved dividing each code group into subgroups from which condensates were developed and rewritten into illustrative quotes. The quotes represented a sum of the participants’ voices from the data. The condensations were checked against the transcripts, to ensure that the condensations represented the participants’ experiences. In the fourth and final step, the condensates were synthesized into an analytical text, as presented in the results. Verbatim quotes of the participants’ own speech were used to illustrate the analytical text, with each participant given a number from 1 to 13 to preserve anonymity. The first and last authors participated in all parts of the analysis, and separately read and coded the data. Themes and coding were thoroughly discussed several times. The final main themes with corresponding sub-themes are presented in Table 2.

**Table 2.** Overview of the Main Themes, Sub-Themes, and Theme Descriptions.

Main Themes	Sub-Themes	Theme Descriptions
Critical period after being reported to the police	*Feeling left alone in distress* *Unhelpful professional responses* *Helpful professional responses*	Experiences of losing it all, standing alone both practical and emotional Being pre-judged, lack of continuous information, negative attitudes Met with humanity, kindness and actions that shows respect and care
Health care intervention	*The importance of a trusting alliance* *Holistic approach*	Met in an open and non-judgmental manner, to be taken seriously in a safe context Focus on the causes of anger problems, and the big picture, further referral to relevant services
Violence desistance: Emotional and practical needs	*Immediate access to support* *Providing hope and trust* *Multidisciplinary help and support*	In need of information about the criminal proceedings and accessible health- and social services Met as a human being, be seen, listened to, given hope and met with trust Over time, like for example, anger management, mental health, social services, substance abuse treatment, help with the relationship

The COREQ checklist (Tong et al., 2007) was consulted throughout the study to maximize the trustworthiness and credibility of the results. Quotes were translated into English for this report by a native English speaker.

### Ethics

All participants received written and oral information about the study and gave their written consent to participate before the interview started. They were given an opportunity to withdraw from the study at any time. The Regional Committee for Medical and Health Research Ethics in Central Norway concluded that the project was not covered by the objective scope of the Health Research Act and not in need of approval before implementation and publishing (no. 2018/639). The need for consent was waived by the office of the Public Prosecutor for Norway, the Norwegian Police Directorate and the Norwegian Social Data Services. The study met the requirements of the General Data Protection Regulation (GDPR) and a Data Protection Impact Assessment (DPIA) was conducted (no. 59923).

## Results

Some of the interviews were conducted shortly after the perpetrator was reported to the police, while others were interviewed several months later. This means that the participants had somewhat different experiences concerning the onset of help and support from the High-Risk Team depending on how this onset date related to the interview date. The three overarching themes into which the findings were categorized were: (a) Critical period after being reported to the police; (b) Health care intervention; and (c) Violence desistance: Emotional and practical needs.

Each overarching theme consisted of sub-themes, as presented in Table 2.

### Critical Period After Being Reported to the Police

This theme captured the time between the IPV incident and contact with the High-Risk Team. Most of the participants gave detailed descriptions of the difficulties they encountered in the first days, weeks, and, for some, even months after the reported incident. Further, they reflected on factors that intensified or reduced the feeling of being left alone in a state of practical and emotional chaos.

#### Feeling Left Alone in Distress

Those who had a no-contact order as a result of the incident experienced practical challenges through losing access to their home: They felt like they were standing alone on bare ground and dependent on luck and goodwill from their own network to get a roof over their head. After the interrogation or release from custody, they received little help and information from the police concerning available support and practical assistance.

When I came out of custody, it was completely surreal. . . you take everything in, and then you’re standing there. . . what do you do? You’re standing outside the prison walls. . . you have a black binbag . . . and then you’re supposed to. . . where do you go from there? There is no one to take care of you, not then and there. . . if you’re released during the day, then the night will come after all. . . (#4)

Participants described that they were largely left alone by services, isolated and with a total experience that everything was falling apart and that they had lost everything. Lack of information, not having any perspective on how long the criminal justice process will last and not knowing what will happen next, were all experienced as a profound psychological strain. Most of the participants said that they did not receive information about available support in relation to the emotional distress they experienced.

Lack of information and a lack of time perspective about the proceedings, the charges, their relationship with their partner and/or children, practical aspects of the future (i.e., finances, job, house) on top of having no one to talk to about these things, led to a strong feeling of being pre-judged and emotional chaos. The situation resulted in different reactions among the participants; some experienced strong suicidal thoughts, others lost trust in the police and felt frustrated and angry. Some of the participants described this period as critical for the avoidance of new violence.

So, at high risk. . . it’s probably from the moment you’re released from the cell and out on the street until at least the interrogation is over (#7)

For some, the emotional chaos made it difficult to understand and adhere to forms describing restrictions imposed on them, that is, a no-contact order:

They explained everything, but you know, the next day. . . I don’t remember. And I started writing text messages . . . for me, it all happened in a big chaos, everything, going to trial and all. . . and this paper doesn’t matter to me, I can just throw it away. . .. (#2)

#### Unhelpful Professional Responses

The experience of being pre-judged by the police officers is repeated by most of the participants. They expressed a strong need to be listened to and given a chance to explain their side of the story/incident. However, the initial contact with the police intensified the emotional chaos and left no hope for the future. Several asserted that the police were biased and had a clear victim focus. Furthermore, they said that the police largely emphasized the victim’s side of the story, and thus were less concerned with elucidating the whole picture of the incident.

. . .It’s about being able to talk to people. . . and being supportive to people. . . They should also talk to each party. . . and I think the police could have been more open. . . where you can tell them what has happened and that you also need help. . . be more supportive and the first point of contact to assist. . . because they have a greater ability to refer you further (#10)

#### Helpful Professional Responses

Despite the fact that the majority described difficult encounters with the police in the time after the report of the incident, they also described being met by the police in ways that made the situation bearable. Some emphasized that to be met with empathy and humanity was an important contribution enabling a sense of hope. For instance, they valued meeting someone who was nice and likeable, who gave a pat on the back and who generally treated them with kindness. Others described respectful and caring actions by the police. This was elucidated by the experience of someone who listened and asked if they needed help. Moreover, it was felt important that the police officers appeared open minded and non-judgmental by being understanding and answering questions.

. . .there was someone who asked if I wanted something. I got a cup of coffee, and then he stood there and talked with me a bit, trying to connect with me in a way. He could see that I was in a quite vulnerable position. So he tried to take care of me a bit. It was very nice to have someone who maybe took a little time then. (#13)

Although most participants described the first period as difficult, only a few participants realized that they needed help and contacted the GP on their own initiative. One of these people was referred to the local mental health care service in the municipality, while others were only prescribed tranquilizers/sleeping medication. In general, they expressed little confidence in the health- and social support system due to previous experiences of being rejected, or not taken seriously. Also, it is important to note that the quality of the initial contact with the police appeared to be important for the degree of motivation and interest in meeting with the High-Risk Team.

### Health Care Intervention

Most of the participants appreciated that the police suggested assistance from the High-Risk Team and other specialized services for perpetrators of IPV. A few were sceptical because of previous negative experiences with the police, but they still gave it a chance.

. . .I don’t think I would have thought about anger management myself back then. . . but if that had been the case, it probably would have just remained a thought. . . But in a way, it was fine that. . . they actually came to our house. . . just one day. . . and talked about it there. . . yeah. (#5)

#### The Importance of a Trusting Alliance

The participants highlighted the importance of being met with openness and non-judgmental attitudes from the police and health- and social workers. They particularly emphasized the value of someone being willing to listen to them, being supportive, and being interested in their situation and what they were struggling with. To be taken seriously and to be met with understanding in a safe context were crucial for further contact. Several spoke about fundamental problems with distrust towards others, and the High-Risk Team’s focus on establishing a trusting alliance was emphasized as important. It increased the participants’ confidence that someone was able and willing to help.

. . .the only thing I need is for them to listen to me and understand my problems. . . so that they help me understand. . . .and ask me the right questions afterwards. That’s what I need. Just to get my head straight. (#8)

#### Holistic Approach

Most of the participants had previous experiences with “silo mentality” within the support system, where mental health problems, substance abuse, and trauma were seen in isolation and separated from anger problems. They found it positive and useful that the High-Risk Team focused on the totality and had a holistic approach to individual needs and the causes of their anger problems, not just a narrow focus on the actual IPV incident and their violent behavior.

I felt like they were trying to understand my situation and hear about what I was struggling with. I was able to explain to them a bit about how I experienced it, and. . . at the last meeting, they conducted a test on me to find out if I was depressed. . . And then it was discovered that there were other things from my childhood. . . and that it was easy to turn to alcohol when things piled up. (#3)

They appreciated that the High-Risk Team recognized their need for persistent, multidisciplinary support going forward, by establishing contact with, for example, the health care system, the GPs, the family welfare office, and social services.

We talked about trying to bring everyone together, and I think that’s good because I talk a little bit here and a little there. . . so maybe no one knows exactly what we’re talking about. And if you get people together, then I think there will be more substance to it. (#4)

### Violence Desistance: Emotional and Practical Needs

All participants emphasized the importance of early support and help from the police in order to prevent future violence towards partner or self. They needed continuous information from the police about the criminal case process and highlighted that the police should work to ensure that the information was understood.

#### Immediate Access to Support

The participants underlined the need for information from the police about accessible health- and social services for perpetrators. Although the participants were offered help from the High-Risk Team, they felt that this should have been offered immediately after being reported to the police. Moreover, they suggested that in addition to existing services, low-threshold and cost-free services should be available for perpetrators.

. . . the need to talk to someone, a phone number or an address. . . an offer where I could talk. Some sort of crisis center or crisis hotline on the same day. That someone reaches out, I wouldn’t have taken the initiative myself. It would have been nice if a friendly voice had called asking, how are you doing, what are you thinking? Someone who isn’t judgmental. (#7)

#### Providing Hope and Trust

The perpetrators were in a chaotic mental state after being reported to the police. Many had not spoken to their partner or children after the violent incident, and all of their work, financial, and housing conditions were unpredictable. Being in such an emotionally and practically difficult situation, the participants underscored how important it was to be seen not only as a perpetrator of IPV but also as a human being. Finally, the participants described the importance of providing hope for the future, both from the police and the High-Risk Team:

It’s nice to have someone to talk to, I’ve never had that. We’ve talked about life and about the way forward and what has happened and what’s ahead and things like that. And then . . . I feel like I’m being believed. (#11)

#### Multidisciplinary Help and Support

The interviews had no specific focus on details concerning the actual reported IPV incident, nor on previous history, but most of the participants spoke spontaneously about difficult growing up conditions, characterized by substance abuse by carers and/or themselves, being subjected to violence during their childhood, mental health challenges and long-term anger problems. They wanted easier access to health services, relating to identification and treatment of mental health problems. Several called for low-threshold and cost-free anger management treatment. Further, participants wanted help with communicating with their partner. Some had earlier attended anger management treatment, and interestingly, some participants suggested couples’ therapy as a viable intervention. Among those who were still in a relationship with the victim, some expressed a need for a common arena where they both got help and support in dealing with challenges in the relationship, as well as challenges related to children, family finances, etc.

We are both interested in making the relationship work. If we’re going to make it succeed, we have to try to do it properly, and both of us must put in the work. Something along the lines of couples therapy and talk therapy. . . It is communication that is the problem. And that is the thing we need to work on together. It’s about understanding the other party and being able to communicate in a normal way. . . that’s what most arguments start with. (#11)

## Discussion

The overall objective of this study was to explore how perpetrators of severe IPV experienced the period following being reported to the police and offered help from professionals. The qualitative analysis identified important barriers in, and facilitators of, the interaction with a multidisciplinary High-Risk Team after the violent incident had occurred.

The perpetrators described a difficult and demanding time after being reported for IPV, and most of them felt they had no one to turn to for support during this critical period. Our findings are in line with previous research reporting the importance of professionals being able to support perpetrators of IPV in a non-judgmental manner as part of the process of enabling them to take responsibility for the violence (Sengoelge et al., 2021).

The perpetrators in our study described how their life had changed radically from one day to the other following being charged; from being a husband or wife, a father or mother, and an income provider, some were not allowed to contact their family, some were incarcerated and others were just left alone with no one to talk to. Importantly, this sudden change in life situation constituted a crisis and caused high emotional distress for the perpetrators. Such stressful circumstances have been linked to the risk of further IPV and hence should be addressed by the police and health- and social services as part of the prevention plan (Arvidsson & Caman, 2024; Breet et al., 2019). Also, many perpetrators of IPV have childhood experiences of violence from family members (Petruccelli et al., 2019; Syed et al., 2023), which in adulthood is associated with psychosocial challenges like anxiety and depression, substance use, as well as disorders involving poor impulse control (C. Spencer et al., 2019). A significant contribution to existing knowledge is that our findings elucidated a great need of continuous information from the police about the criminal case process, which may have reduced the level of perpetrator distress and in turn potentially reduced the risk of impulsive violence towards the victim partner or self.

Lack of motivation to accept and be subjected to professional help has been identified among perpetrators of severe IPV (Lila et al., 2019; Petersson & Strand, 2017), and the police have a great opportunity to start a motivating process towards accepting help from health- and social workers, for example, the High-Risk Team, during their initial contact with the perpetrators. Another barrier to the perpetrators realizing that they have a violence problem is the difficulty to verbally describe the violent behavior (Sengoelge et al., 2021). Hence, it seems pivotal to be offered help from professionals with competence in how to talk about violence and who encourage the verbal expression of difficult emotions (Arvidsson & Caman, 2024). However, as treatment alone has yielded inconsistent results in ending violent behavior among high-risk perpetrators of IPV, our study is in line with a growing interest in exploring alternative interventions involving professionals across agencies working in partnership as in this High-Risk Team (e.g., police, health- and social services) (Juodis et al., 2014; Notko et al., 2022).

By working in a multi-disciplinary way, it is possible to provide practical and emotional support to both of the involved parties in a manner that de-escalates the risk (Juodis et al., 2014; Notko et al., 2022). In our study the perpetrators and their victims (partners and children) were offered help from a multi-professional High-Risk Team of nurses, psychologists, social workers and police officers. The High-Risk Team used a conceptual framework recognizing the psychological, economic, social and environmental impact of IPV. Violence risks and needs were assessed and tailored interventions were offered accordingly. For instance, many victims and perpetrators would try to contact each other after a report to the police about IPV, even if there was a no-contact order between them. This may pose a heightened risk for fatal incidents occurring and is a highly undesirable situation. Victims and perpetrators failing to comply with protective restrictions imposed on them by the police, make it hard for the police alone to ensure safety for the victims.

Time was also highlighted by the perpetrators as an important dimension, and many described how they would have liked to be offered someone to talk to immediately after being reported to the police. They described how they experienced their life as being in limbo after being reported to the police, not knowing what would happen next. To be offered someone to talk to immediately after being reported to the police would possibly have reduced the internal stress they experienced.

Learning how to communicate better about violent situations and partner conflicts was important to the perpetrators in our study, and some suggested couples’ therapy as a relevant intervention. Researchers have found that under certain circumstances couples’ therapy may be just as effective as, for instance, group therapy for perpetrators only (Brannen & Rubin, 1996; Portnoy & Murphy, 2020), and in some cases may even be more effective in reducing violence (Mills et al., 2019).

### Strengths and Limitations

A strength of the current study was the study design which allows the participants to describe and explain their own experiences and preferences. The study used an exploratory design with a heterogeneous sample to uncover different experiences. Both the preliminary results and the analysis were discussed among the authors to strengthen the interpretations and to validate the findings. Our study participants were a self-selecting sample and varied with regard to sex, age, and mother tongue, as well as socio-economic characteristics like education and work status. However, due to the sensitivity of the results, we refrained from presenting results from subgroups. Future research could benefit from focusing on female perpetrators, male victims, culture and same sex relationships in order to obtain a deeper understanding of needs of diverse cultural and gender groups. Our study had some limitations that may have affected the results. Time between the IPV incident and first contact with the High-Risk Team varied between the participants. Some received assistance after just a few days, while some had to wait several months. Those who had to wait a long time before receiving the intervention may potentially have had different experiences compared to those who got help immediately from the High-Risk Team. High workload among investigators prolonged the investigation process, and thus some of the perpetrators were offered health- and social care later rather than sooner. This variation may have influenced the perpetrators’ experiences as reported to the researchers. In addition, some of the interviews were conducted shortly after the perpetrator was reported to the police, clearly a time of high distress, while others were interviewed several months later. This variation might also have influenced the responses made to the interviewers. Although we could not identify a different pattern of response among those interviewed early versus later, future research may want to explore this further.

Also, it must be acknowledged that the sensitivity of the topic and the fact that the perpetrators were under investigation by the criminal justice system at the time of the interview may have influenced their willingness to disclose important aspects about the violent incident and their experiences subsequently.

## Conclusions

The exploratory inductive design of this study enabled participants to provide rich in-depth descriptions of their experiences without researcher assumptions about what was likely to be relevant. The sample contained a good mix of perpetrators in terms of working situation, relationship status (terminated or otherwise), age, and the involvement of children. There was representation of non-Norwegian and female perpetrators. In conclusion, the IPV perpetrators in this study seemed to benefit from the provision of a multidisciplinary High-Risk Team in the chaotic period following police intervention. They were enabled to stabilize and gain some insight into their behavior which is likely to contribute to minimizing further incidents. However, this is only a small sample of perpetrators of IPV, who all consented to get help from the High-Risk Team. Hence, the study has no information about those refusing to accept assistance from the team. Nevertheless, the study has provided some important insights, which should inform the future design of IPV prevention services.

## Supplemental Material

sj-docx-1-jiv-10.1177_08862605251355622 – Supplemental material for A Multi-Disciplinary Approach to Intimate Partner Violence: A Qualitative Study of the Perpetrators’ Experiences Supplemental material, sj-docx-1-jiv-10.1177_08862605251355622 for A Multi-Disciplinary Approach to Intimate Partner Violence: A Qualitative Study of the Perpetrators’ Experiences by Camilla Buch Gudde, Tom Palmstierna, Richard Whittington and Merete Berg Nesset in Journal of Interpersonal Violence

sj-pdf-2-jiv-10.1177_08862605251355622 – Supplemental material for A Multi-Disciplinary Approach to Intimate Partner Violence: A Qualitative Study of the Perpetrators’ Experiences Supplemental material, sj-pdf-2-jiv-10.1177_08862605251355622 for A Multi-Disciplinary Approach to Intimate Partner Violence: A Qualitative Study of the Perpetrators’ Experiences by Camilla Buch Gudde, Tom Palmstierna, Richard Whittington and Merete Berg Nesset in Journal of Interpersonal Violence
