# Supplementary material for: A Multi-Disciplinary Approach to Intimate Partner Violence: A Qualitative Study of the Perpetrators’ Experiences
Source: J Interpers Violence. 2025 Jul 15;41(15-16):5348–69. doi: 10.1177/08862605251355622 (PMC13373290; doi:10.1177/08862605251355622)
Supplement: sj-docx-1-jiv-10.1177_08862605251355622 – Supplemental material for A Multi-Disciplinary Approach to Intimate Partner Violence: A Qualitative Study of the Perpetrators’ Experiences [file sj-docx-1-jiv-10.1177_08862605251355622.docx]

**Additional file 1**

Original Semi-structured interview guide for individual interviews with perpetrators of intimate partner violence

1. **Experiences from meeting the police – the current incident**

Can you tell us about:

- The first contact with the police?
- Information regarding the case?
- What did you need in the situation? (practical, emotional?)
- Information regarding were to find support/help?

1. **Experiences from protective measures**

(If yes) Can you tell us about:

- What type?
- Challenges in complying? (Why or why not?)

1. **Experiences from general health-/social services**

Can you tell us about:

- Your contact with health- social services (e.g. GP, social welfare)
- What kind of help/support are important for you?

1. **Experiences from the High Risk Team**

Can you tell us about:

- When you received information about the High Risk Team?
- What made you say yes to participate?
- What has been it relevant/important to you?
- What has been less relevant/important to you?
- Anything you missed?

1. **Thoughts about violence prevention**

Can you tell us about:

- What is most important for you to stop acting violent in the future?
